# Individualized Meal Replacement Therapy Improves Clinically Relevant Long-Term Glycemic Control in Poorly Controlled Type 2 Diabetes Patients

**DOI:** 10.3390/nu10081022

**Published:** 2018-08-04

**Authors:** Kerstin Kempf, Martin Röhling, Katja Niedermeier, Babette Gärtner, Stephan Martin

**Affiliations:** 1 West-German Centre of Diabetes and Health, Düsseldorf Catholic Hospital Group, Hohensandweg 37, 40591 Düsseldorf, Germany; kerstin.kempf@wdgz.de (K.K.); katjaniedermeier@web.de (K.N.); babette.gaertner@vkkd-kliniken.de (B.G.); stephan.martin@vkkd-klinken.de (S.M.); 2 Faculty of Medicine, Heinrich Heine University Düsseldorf, 40225 Düsseldorf, Germany

**Keywords:** type 2 diabetes, low-carbohydrate diet, HbA1c, weight loss, formula diet

## Abstract

*Background* Formula diets can improve glycemic control or can even induce remission in type 2 diabetes. We hypothesized that especially an individualized intense meal replacement by a low-carbohydrate formula diet with accompanied self-monitoring of blood glucose (SMBG) contributes to long-term improvements in HbA1c, weight, and cardiometabolic risk factors in poorly controlled type 2 diabetes. *Methods* Type 2 diabetes patients were randomized into either a moderate group (M-group) with two meal replacements/day (*n* = 160) or a stringent group (S-group) with three meal replacements/day (*n* = 149) during the first week of intervention (1300–1500 kcal/day). Subsequently, both groups reintroduced a low-carbohydrate lunch based on individual adaption due to SMBG in weeks 2–4. After week 4, breakfast was reintroduced until week 12. During the follow-up period, all of the participants were asked to continue replacing one meal per day until the 52-weeks follow-up. Additionally, an observational control group (*n* = 100) remained in routine care. Parameters were compared at baseline, after 12 and 52 weeks within and between all of the groups. *Results* 321 participants (83%) completed the acute meal replacement phase after 12 weeks and 279 participants (72%) the whole intervention after 52 weeks. Both intervention groups achieved improvements in HbA1c, fasting blood glucose, blood pressure, and weight (all *p* < 0.001) within 12 weeks. However, these results were not significantly different between both of the intervention groups. The estimated treatment difference in HbA1c reduction was (mean (95% confidence interval [CI]) -0.10% with 95% CI [−0.40; 0.21] also (*p* > 0.05) (S-group vs. M-group) not statistically different after 12 weeks. However, only the S-group showed a clinically relevant improvement in HbA1c of −0.81% [−1.06; −0.55] (*p* < 0.001) after 52 weeks of follow-up, whereas HbA1c was not statistically different between the M- and control group. *Conclusion* Individualized meal replacement with SMBG demonstrated beneficial effects on HbA1c and cardiometabolic parameters in type 2 diabetes. Furthermore, the initiation of a weight loss program with one week of full meal replacement (three meals per day) resulted in a clinically relevant long-term HbA1c reduction, as compared to an observational control group that had standard care.

## 1. Introduction

Current type 2 diabetes mellitus guidelines recommend lifestyle intervention as basic treatment. However, patients often fail to improve their eating behavior, physical activity, body weight, and glycemic control in the long run. In this context, new strategies have been developed, such as technology-based approaches [1], to improve adherence to lifestyle interventions and to enable long-term benefits [2]. In contrast, a failing lifestyle intervention contributes to an initiation of a pharmaceutical co-intervention in the next step, however, anti-diabetic medication does not prevent the progression of type 2 diabetes [3]. Within 10 years after diagnosis, about 50% of type 2 diabetes patients start with insulin therapy [4]. This often results in additional weight gain, leading to an increased insulin dosage [5]. Thus, this vicious circle proceeds and disease remission had been unlikely until bariatric surgery demonstrated that type 2 diabetes is reversible [6]. After bariatric surgery, glycemic control improves within a few days, even before a decrease of body weight becomes apparent [7], but this treatment has several severe side-effects [8] and long-term effects are still unclear [9]. In this context, it is still unknown whether the magnitude of improvement is primarily due to caloric restriction or is unique to the surgical procedure [10]. Given the huge need for alternative approaches with long-term effects regarding HbA1c reduction and remission of diabetes, formula diets can be simple and effective measures [11]. Furthermore, the use of energy-restricted formula diets in obese persons with type 2 diabetes improved cardiometabolic endpoints, e.g., waist circumference, fat mass, blood pressure, insulin, or HbA1c, [12]. Moreover, intervention studies, especially those from a group in the United Kingdom (UK) [11,13,14,15], with a stringent and very low-calorie formula diet were even able to induce diabetes remission [13,14,15]. In previously published studies, we had already investigated the single or combined effect of low-carbohydrate formula diets and/or telemedicine in patients with type 2 diabetes inducing HbA1c, anti-diabetic medication, and body weight improvements [12,16]. Furthermore, we could also demonstrate the beneficial effect of individual meal prescription accompanied with self-monitoring of blood glucose (SMBG) in patients with type 2 diabetes [17]. However, there are hardly any studies investigating the dose-response relationship of an early intense and individualized low-carbohydrate and moderate-calorie meal replacement therapy by formula diet in patients with type 2 diabetes. Furthermore, a previous study revealed a high dropout rate of 32% for a stringent diet intervention with low-carbohydrate meal replacement [12]. We, therefore, conducted the current intervention by comparing two diet regimens, differing in treatment intensity, with a third observation control group that remained in routine care, in patients with type 2 diabetes.

## 2. Materials and Methods

### 2.1. Study Design

The present study consisted of two intervention groups and one observational control group. Volunteers were recruited in Germany by newspaper articles. Eligible type 2 diabetes patients were randomized according to an electronically generated randomization list into two parallel intervention groups with either a moderate (M-group, *n* = 160) or a stringent diet regime (S-group, *n* = 149). The observational group (*n* = 100) corresponds with the control group from our TeLiPro study (NCT02066831) [16]. The participants, the study nurse, and the outcome assessor were blinded for sequence of allocation concealment. The first participant was enrolled on 7 February 2012 and the last participant finished the intervention on 13 June 2014. The study was conducted at the West-German Centre of Diabetes and Health in Düsseldorf (WDGZ), Germany, in cooperation with family doctors and diabetologists around Germany and in accordance with the ethical standards that were laid down in the 1964 Declaration of Helsinki and its later amendments. Approval of the research protocol was obtained from the ethics committee of the Ärztekammer Nordrhein (No. 2011294) and it was registered at clinicaltrials.gov under the number NCT02230501, ClinicalTrials.gov. All of the participants gave written informed consent prior to their inclusion into the study.

### 2.2. Study Population

Patients with type 2 diabetes, aged 25–79 years with poorly controlled glucose levels (HbA1c ≥ 7.5%), and body mass index (BMI) ≥ 27 kg/m^2^ were included in the study. Participants were excluded when one of the following exclusion criteria was existent: (i) acute infections; (ii) chronic diseases such as cancer, chronic obstructive pulmonary disease, asthma, dementia, chronic gut diseases, psychoses, liver cirrhosis, nephropathy, and kidney insufficiency with glomerular filtration rate < 30 mL/min/1.73 m^2^; (iii) weight loss of >2 kg/week in the last month; (iv) smoking cessation or planned smoking cessation during the study; (v) drugs for active weight reduction; (vi) pregnancy or breast-feeding; and, (vii) known intolerance with components of the used formula diet.

### 2.3. Intervention

At the first contact, the design and intention of the study were explained to the participants by study nurses and trial physicians. A manual and a formula diet were handed out to the patients of the intervention groups. The manual included information about the preparation of the individualized meal replacement as well as general facts about low-carbohydrate meals and their interaction with the blood glucose level. Participants were instructed to perform self-monitoring of blood glucose (SMBG) and note down these values into the manual, the amount of meal replacement taken, the number of meals replaced, as well as their daily dose of anti-diabetic medication. Participants were advised to perform a seven-point blood glucose diurnal profile and they were urged to perform event-driven measurements, e.g., 1.5–2 h after no, low-, or high- carbohydrate consumption or in the fasting state in the morning when exercise had been done the evening before. The patients were encouraged to draw their own conclusions from the SMBG results and to adapt their meals and habits aiming to keep blood glucose levels within a normal range, which was individually prescribed and adapted during the study process. The manual provided guidance on how to change eating habits and how to react to elevated blood glucose levels with physical activity. Based on their own experience and in accordance with the prescriptions to adapt their blood glucose levels, participants were responsible for modifying their diet and received help in the case of nutrition-related uncertainties. In sum, meal replacement and SMBG were individually recommended and adopted to the personal preferences throughout the study. Based on these values, anti-diabetic therapy was monitored and then individually adjusted by trial physicians. This “personalized nutrition and treatment” was one of the main educative approaches in our study. At each visit, study nurses revised the manual and educated/instructed the participants in terms of low-carbohydrate diet, SMBG, physical activity, and self-motivation. Study visits took place after week 1, 4, and 12 and were accompanied with telephone calls or personal meetings. A detailed timeline of the study visits is shown in the Supplementary Figure S1. Participants of the control group only received a self-management guide, a weighing scale, as well as a step counter and they were advised to measure their steps and weight daily.

### 2.4. Outcomes and Measurements

Clinical and biochemical data were measured at baseline, after 12 weeks of intervention, and after 52 weeks of follow-up. Venous blood was collected after an overnight fast and abdication of medication of at least 10 h by inserting an intravenous cannula into the forearm vein, and laboratory parameters (HbA1c, fasting blood glucose, total cholesterol, high-density-lipoprotein (HDL), and low-density-lipoprotein (LDL) cholesterol) were analyzed at the local laboratory as described in detail elsewhere [16]. Validated questionnaires were used to assess eating behavior (German version of the ‘Three-factor Eating Questionnaire’ (TFEQ)) and quality of life (‘Short Form-36’ (SF36)), as previously described [16]. Anti-diabetic medication and changes throughout the study were documented. Adverse events were documented.

### 2.5. Diet Regimen

The chosen formula diet (Almased-Vitalkost; Almased-Wellness-GmbH, Bienenbüttel, Germany) contained 30.6 g carbohydrates and 1507 kJ (360 kcal) energy per 100 g powder and it was provided to all study probands during the whole study period. Participants of the intervention groups replaced breakfast, lunch, and dinner with 1 g Almased/kg normal body weight (defined as height in cm −100) per meal dissolved in 250 mL water during the first week and consumed 45 g of oil rich in omega-3-fatty acids (1665 kJ; 398 kcal) and 750 mL vegetable juice each day, as previously described [16]. No additional snacks were permitted. During weeks 2–4, the participants replaced breakfast and dinner with the formula diet and ate a low-carbohydrate lunch. The lunch should include 150–200 g of fish or meat, 500 g vegetables, and not more than 50 g of carbohydrates from wholegrain bread or brown rice. The low-carbohydrate nutrition had to be continued in the weeks 5–12, while only dinner was replaced by formula diet. Instructions were identical for the participants of both groups. The only difference between both intervention groups was that the M-group should only replace two meals per day during the first week. All of the participants were asked to continue replacing one meal per day during the follow-up period until the final visit at the 52-weeks follow-up. Both participant and study staff were responsible for the individualized treatment. SMBG as well as the personalized formula diet and the reintroduction of normal meals were interactively modified. Furthermore, the personalized formula diet depends on the current weight of each proband and is characterized by low-carbohydrate meals that are aiming to regulate a normal blood glucose level. We assessed protocol compliance by requiring the participants to note the frequency and amount of formula diet they used as well as the composition of their meals during the first 12 weeks. This information had to be sent back. Afterwards, they got another ration of formula diet for the next weeks. We chose this design with a very similar intervention program as the current study situation reveals that only intense behavioral lifestyle interventions can contribute to meaningful results [14], and we were interested in the dose-response pattern in initial treatment phase during the first week. Furthermore, we had seen in a previous study that a very stringent regime leads to high dropout rates, and we, therefore, wanted to test a gentler entry [12]. The control group remained in routine care (quarterly visits with their attending physician for routine health-care visits, as defined by the Disease Management Programs (DMP) for Type 2 Diabetes in Germany) and did not participate in the meal replacement program.

### 2.6. Statistics

Previous own data have indicated that with the use of a low-carbohydrate meal replacement a reduction in HbA1c of 0.7% could be achieved [12], while a reduction of 1.0 ± 0.8% for the S-group was assumed. To be able to measure differences between both of the intervention groups with a power of 80% and a level of significance of 5%, a sample size calculation revealed that at least 230 datasets would be needed. Since a dropout rate of about 25% was estimated, the plan was to recruit a total of 140 participants per group. Data are presented as means and standard deviations (mean ± SD), median and first and third quartiles (median (first; third quartiles)), means and 95% confidence intervals (mean [95% CI]), or percentages, as appropriate. Completer analyses were performed. Missing values were imputed by the ‘last-observation-carried-forward’ (LOCF) principle. As HbA1c is the primary parameter in the present study, LOCF was solely applied for other parameters. 

Primary endpoint was the differences in Hba1c after 12 weeks between groups, secondary outcomes were the differences in body weight, BMI, cardiometabolic risk factors, eating behavior, quality of life, and frequency of anti-diabetic medication after 12 weeks of meal replacement intervention and 52 weeks of follow-up between the two intervention groups. Furthermore, the estimated treatment difference (ETD), as well as the proportion of weight loss in percentage, was determined. Non-parametric data were analysed with Mann-Whitney U, Wilcoxon, and Friedman test and parametric data with Student’s *t*-test, paired *t*-test, and analysis of variance with repeated measures to determine the differences between groups following the intervention. Multivariable univariate regression analyses were carried out to investigate group differences while adjusting for baseline parameters. Dichotomous variables as well frequencies were compared by the Fishers exact test, McNemar test, or Cochrane Q test.

Tertiary outcomes focused on changes in all aforementioned parameters from baseline to week 12 and week 52 within both intervention groups. These were analyzed while using mixed models adjusting for repeated measurements, baseline values, and multiple testing.

Further analyses focused on differences between the intervention groups and the observational control group in regard to HbA1c and weight loss. These analyses were performed in accordance with the statistical approaches used for the determination of the primary endpoints. All statistical tests were two sided, and the level of significance was set at α = 0.05. *P* values were adjusted for multiple comparisons using Bonferroni correction. All of the analyses were performed using SPSS 22.0 (SPSS Inc., Chicago, IL, USA) and GraphPad Prism 6.04 (GraphPad Software, San Diego, CA, USA).

## 3. Results

**Figure 1:**
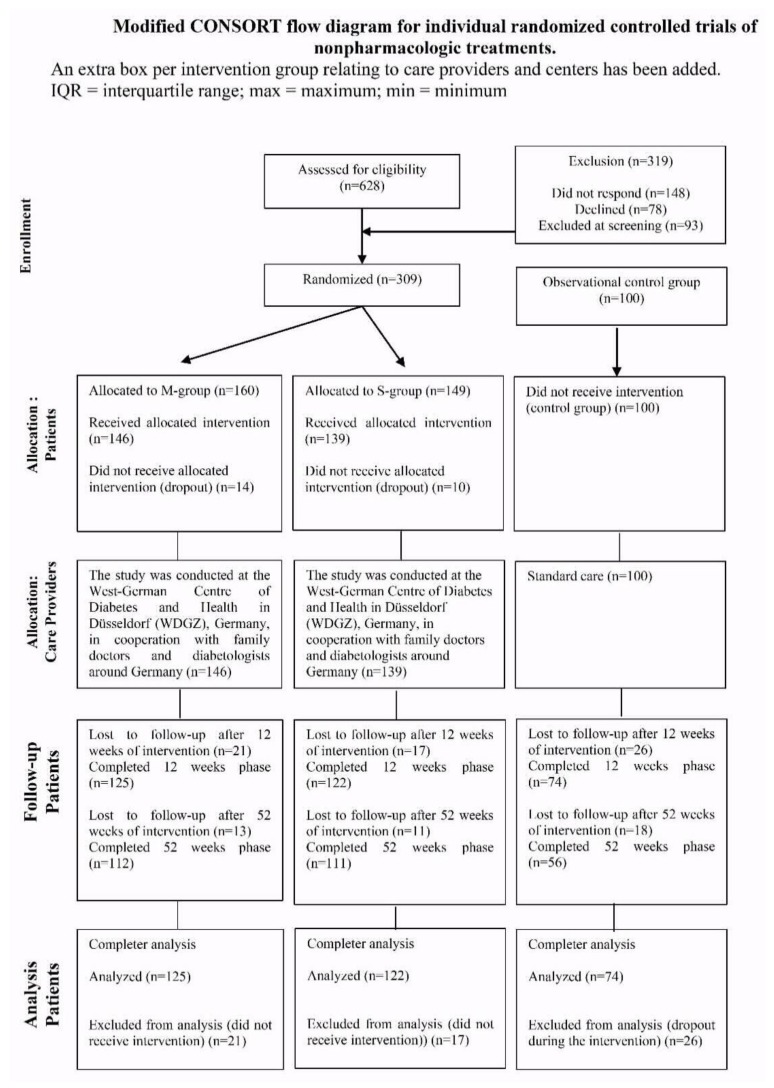
Flow chart.

A total of 309 participants were randomized into the S-group (*n* = 149) or M-group (*n* = 160), and a control group of *n* = 100 were observed, as shown in Figure 1. Three hundred and twenty-one participants (83% [321:385], *n* = 125 M-group; *n* = 122 S-group; *n* = 74 control group) from the starting cohort finished the 12-weeks intervention, while 64 participants dropped out within the 12-weeks period. Follow-up data after 52 weeks were available from 279 participants (72% [279:385]). Reasons for dropouts were: (i) spontaneous intolerances (5%); (ii) health problems (25%); (iii) professional reasons (5%); (iv) personal reasons (60%); and, other reasons (5%). The demographical and clinical characteristics of the three groups are shown in Table 1. Participants who completed the intervention and follow-up phase and those who dropped out or were lost to follow-up did not differ significantly, apart from differences in diabetes duration, eating behavior, and quality of life between the groups (Supplementary Table S1). No adverse effects have been reported. Patients of the control group were more frequently treated with antidiabetic medication than those in the intervention groups, particularly, regarding insulin therapy. The individual antidiabetic drug classes are listed in Supplementary Table S2.

**Figure 2:**
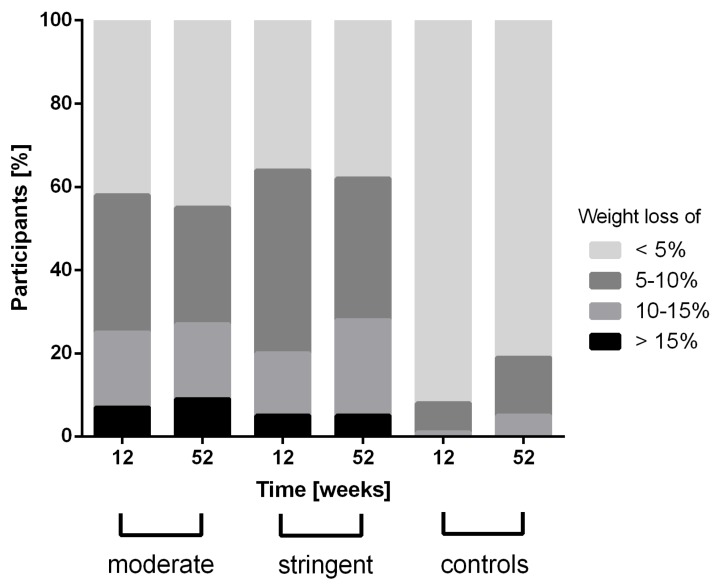
Weight change achieved after 12 and 52 weeks of intervention. Analyses of difference in frequency distribution of weight loss were calculated by using Fisher’s exact test.

Besides marginal differences in eating behavior and triglycerides, both intervention groups showed no significant differences in any parameter at week 12 or 52 (Table 2). The ETD in HbA1c reduction after 12 weeks between both intervention groups was −0.10% with 95% CI [−0.40; 0.21] (*p* > 0.05). Treatment superiority of the S-group vs. M-Group is not statistically significant after the 52-weeks follow-up with −0.22% [−0.56; 0.10] (*p* = 0.15). Furthermore, the proportion of weight loss between both of the intervention groups was not different from baseline to week 12 and week 52 (Figure 2).

After 12 weeks of intervention, HbA1c was reduced by (mean [95% confidence interval (CI)] −0.97% [−1.21 to −0.74] in the S-group and by −0.84% [−1.08 to −0.61] in the M-group (both *p* < 0.001) as shown in Table 3. These improvements were still significant after the Bonferroni correction for multiple testing. After 52 weeks of follow-up, the reduction of HbA1c lost its clinical relevance (≥0.60%) [18] in the M-group with −0.55% [−0.80 to −0.29] when compared to the S-Group with −0.81% [−1.06 to −0.55]. Patients of the control group showed no improvement in HbA1c neither after 12 weeks nor after 52 weeks.

Changes of anthropometric, clinical, pharmaceutical, and behavioral parameters within both of the intervention groups after 12 and 52 weeks of intervention are shown in Table 3. Improvements in body weight, BMI, fasting blood glucose, systolic and diastolic blood pressure, as well as eating behavior were observed in the M- and S-group after 12 and 52 weeks of follow-up (all *p* < 0.01). These changes in HbA1c, weight, BMI, fasting blood glucose, systolic and diastolic blood pressure, as well as eating behavior were still significant after the Bonferroni correction for multiple testing (*p* value = 0.002) in the within-groups analysis. Doses of anti-diabetic medication was already adjusted within the first week of intervention. Frequencies of anti-diabetic drugs were not significantly changed within groups after Bonferroni correction.

**Figure 3:**
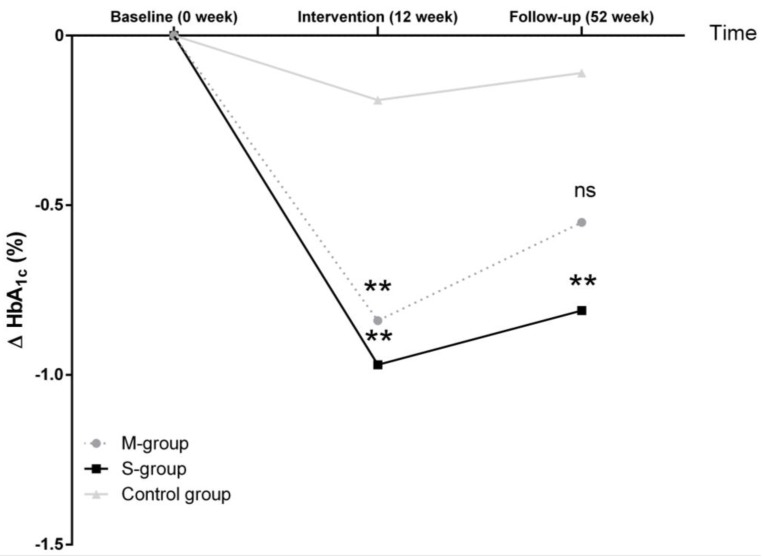
Change of glycemic control after 12 weeks of intervention and 52 weeks of follow-up. At baseline, M-, S- and control group were not significantly different, however, 12 weeks of diet intervention led to reductions in HbA1c in both intervention groups in comparison to the control group. Compared to controls, only the S-group showed a significant difference in HbA1c after 52 weeks of follow-up. Analyses of variance with repeated measures were performed to determine differences between groups; ns, not significant; ** *p* < 0.01 vs. controls.

When compared to the control group (12 weeks: −0.20 ± 0.80 standard deviation (SD); 52 weeks: −0.10 ± 0.90 SD), only the S-group (12 weeks: −0.97 ± 1.18 SD; 52 weeks: −0.81 ± 1.20 SD) demonstrated a significant difference in HbA1c after 52 weeks of follow-up (*p* < 0.01), while the M-group (−0.84 ± 1.14 SD; 52 weeks: −0.55 ± 1.31 SD) was not significantly different (Figure 3). Furthermore, a higher proportion of participants with a larger weight reduction was shown in the intervention groups after 12 and 52 weeks in comparison to the control group (all *p* < 0.001; Figure 2).

## 4. Discussion

The results of the present study demonstrate that an individualized meal replacement therapy starting with intense low-carbohydrate formula diets and SMBG-accompanied reintervention of low-carbohydrate meals lead to clinically relevant improvements in HbA1c after 12 weeks of intervention in patients with poorly controlled long-standing type 2 diabetes. Particularly, patients of the more intense intervention group (S-group) showed long-term clinically relevant improvements after 52 weeks of follow-up as compared to the participants of the moderate intervention group (M-group), although this difference was not statistically significant. Furthermore, the overall dropout rate after allocation into both intervention groups was small (247:285; 13%) and not different (S-group = 12% and M-group = 14%). We hypothesize that the strict rules, the stringent and individual SMBG [17], and the complete replacement of all meals in the S-group during the first week contributed to a subtler change of behavior and higher motivation for the diet, which was shown to be necessary for long-term changes of behavior in high-risk individuals for type 2 diabetes in prior studies [19]. Furthermore, we assume that our personalized nutrition and treatment-approach with a more intense patient empowerment during the first week in the S-group contributed to a long-term difference in HbA1c after 52 weeks of follow-up. The recently published DIRECT study has demonstrated that a strict calorie restriction with only 825–853 kcal per day for 3–5 months contributes to significant improvements of HbA1c (−0.9%) and body weight, and in the further course to diabetes remission after 52 weeks of intervention [13]. However, the formula diet contained proteins and carbohydrates in a ratio of 1:2 [13]. We chose an opposite formula diet that was high in protein, but low in carbohydrates (ratio nearly 2:1) with individualized moderate-calorie supply (1300–1500 kcal/day), because we postulate that a higher amount of carbohydrates would stimulate an increase in insulin release and a decrease in fat burning [20]. Therefore, our strong carbohydrate reduction with an accompanied stepped food reintroduction should lead to long-term benefits like it was shown before in the DIRECT study [13]. Another explanation could be that the S-group was somewhat higher motivated to be physically active due to the complete change of nutrition and behavior, respectively. Previous studies have already demonstrated strong effects on HbA1c through very low-calorie liquid formula diets in small groups of patients with type 2 diabetes (*n* < 30) during an investigation period up to 26 weeks, especially after a short duration of diabetes (<4 years) [11,14,15]. This correlation between diabetes duration and changes of HbA1c after 12 or 52 weeks could be confirmed in our study by the whole intervention cohort (*r* = 0.226 (after 12 weeks) or 0.229 (after 52 weeks); both *p* < 0.001), independently of age.

Our approach of low-carbohydrate meal replacement is based on the recommendations for diets in type 2 diabetes, as well as recently published reviews and meta-analyses [21,22]. Although, a healthy diet is crucial for type 2 diabetes, there still exists controversy in the field about the feasibility and mechanisms of these stringent types of dietary interventions and their long-term effects in HbA1c [23]. The effects on the glucose metabolism (e.g., anti-diabetic medication was adjusted within the first week) occur immediately after beginning the meal replacement therapy [12] and before a significant weight loss takes place. The observed effects are comparable with those after bariatric surgery [7]. Possible explanation approaches in this context could be altered levels of incretin secretion [24], improved mitochondrial oxidative function [25], energy restriction [10], the sudden negative energy balance [14], or a combination of all these points. Furthermore, a reduced carbohydrate intake [26] or a reduced number of carbohydrate-containing meals might trigger the fast effects on the glucose metabolism. This would be in line with observations that two meals per day are better than six [27] for type 2 diabetes patients, especially in terms of body weight, insulin resistance/sensitivity, and beta cell function [10]. The results of the PREDIMED study, in which two high-fat/lower-carbohydrate Mediterranean diets were compared to a fat-reduced diet regarding the incidence of type 2 diabetes [28] or cardiovascular events [29], as well as changes of body weight and waist circumference [30], support our findings that carbohydrate-reduced diets are beneficial for patients with type 2 diabetes.

The improvements in glycemic control in both intervention groups in the present study were followed by strong reductions in body weight (Figure 2). In a recently published meta-analysis, it was shown that very low-calorie (<800 kcal per day) or low-energy liquid-formula (>800 kcal per day) diets can induce large reductions of body weight (ranging from 8.9 to 15.0 kg) in obese people (BMI: 35.5–42.6 kg/m^2^) with and without type 2 diabetes [31]. The slight difference in body weight reduction in our trial can be explained by a higher calorie consumption per day (≈1300–1500 kcal per day) when compared to the studies of the meta-analysis. Furthermore, our results are comparable to the findings of Steven et al. [11], who works with a very low-calorie and moderate-carbohydrate composition (43% carbohydrate, 34% protein, and 19.5% fat; 2.6 MJ/day [624 kcal/day]). They found that a very low-calorie diet over eight weeks can contribute to a meaningful weight reduction of ≈14 kg, which was still comparably high, even after 26 weeks (≈13 kg) in individuals with type 2 diabetes. In regard to the aforementioned findings, we could demonstrate similar results of weight reduction with ≈7 kg after 12 and 52 weeks of intervention. In contrast to Steven et al., we designed an individualized low-carbohydrate and moderate-calorie diet intervention (31% carbohydrate), accompanied with SMBG as it might be more feasible for patients with type 2 diabetes, characterized with eating and motivation impairments [32]. The improvements, apart from the meal restriction, could be therefore also explained by improved education regarding nutrition, physical activity, and blood glucose control. 

A recently published review supports our approach, as it states that a rather moderate weight loss is more sufficient for the transition from metabolically unhealthy obesity to metabolically healthy obesity with a lower risk for adverse outcomes in the long run than a large amount of weight loss in a short period [33]. We chose this calorie goal per day in order to reduce the rate for dropouts and increase the participants therapy adherence. Lifestyle interventions are always criticized in terms of their long-term effectiveness, and one possible hypothesis says that the major problem is that patients fail to adhere to the altered lifestyle prescriptions [34]. In contrast to many other long-term lifestyle intervention programs [35], the relatively high number of completers after 12 (83%) and 52 (72%) weeks supports our study design and approach. Potential reasons for nonadherence comprise: age, perception and duration of disease, polytherapy, social and psychological factors, costs, dislike for foods included in meal plans, education and a lack of understanding of the long-term benefits of treatment, adverse outcomes (e.g., weight gain or hypoglycemia), as well as negative treatment perceptions [36]. In this context, new innovative methods are needed to assist those patients. In light of these problems, we designed the study with almost no barriers for the participants (e.g., 1:1 personal support or no additional costs) and provided every participant with a personalized meal replacement and supported them in their SMBG.

Further improvements were achieved in the cardiometabolic parameters of fasting blood glucose, as well as systolic and diastolic blood pressure. These results are confirmative regarding other studies with low-calorie diets in patients with a short- and long-duration type 2 diabetes and moderate [14] or poor glycemic control [10,15]. Our results are also confirmed by a recently published review in terms of improvements of the cardiovascular risk profile in patients with type 2 diabetes showing a significant decrease in systolic and diastolic blood pressure as well as fasting blood glucose after low-calorie diets [37].

A further positive effect following the intervention was the improvement of eating behavior in the intervention groups. The simple and structured formula diet reduced feelings of hunger and increased the control regarding eating-associated actions. In patients with type 2 diabetes, a disordered eating behavior can be present and it is associated with poor quality of life [38]. When compared to individuals with the metabolic syndrome, type 2 diabetes participants of the present intervention groups showed a pronounced feeling of hunger and a weaker control over their suggestibility for food [39]. Another study supports our findings showing meaningful improvements in eating behavior after a three-month mindful eating intervention in non-insulin requiring patients with type 2 diabetes in a small cohort (*n* < 30) [40].

A previously published pilot study [12] revealed how a formula diet affects blood glucose control and weight, and how insulin is reduced or discontinued. However, it was also shown that participants sometimes found it difficult to maintain the stringent diet during the first week. Therefore, we were interested in whether a moderate approach also leads to success. The underlying idea was that the replacement of all three meals in the first week would lead to some kind of “reset”. In combination with concomitant blood glucose self-monitoring in the following weeks, an individualized diet should be gradually rebuilt. However, because of the similarity of the intervention design, we expected that the moderate diet regimen would lead to a significant improvement as well. We, therefore, included the comparison with a control group that received standard treatment.

The strengths of the present study comprise: (i) a relatively large number of patients studied per group who had poor controlled type 2 diabetes and a long type 2 diabetes duration; (ii) a longer study period compared to previous studies with formula diets (52 weeks vs. ≤26 weeks); as well as (iii) a randomized trial design with two intervention groups and one observational control group. Furthermore, the (iv) chosen real-world setting with a combination of formula diet, SMBG, and dietary education could be easily implemented into present health care programs. Likewise, another study with a real-world approach could demonstrate that even the partial use of a formula diet with one pack of formula diet instead of one of three daily low-caloric meals for 24 weeks was much more effective in reducing body weight and improving coronary risk factors than a conventional diet with a reduced energy intake in obese type 2 diabetic patients [41].

A limitation of our study is that we did not use food diaries to control for decreased calorie consumption or incorrect food compositions (e.g., the amount of carbohydrate in the diet, glycemic index, fat or protein intake) after the acute meal replacement phase from week 13 to week 52. However, the 52-week follow-up revealed that participants of both intervention groups showed no difference in maintaining the formula diet and following the dietary intervention until the study end (S-group 65%; M-group 63%). Also, more profound and quantitative diagnostics, such as isotope measurements, could have been done to control for food-related study compliance. On the other side, interventional studies with formula diets and similar results in a real-world setting [31] support our therapeutic approach in patients with poorly controlled type 2 diabetes.

Another factor, which should be considered, is the adjusted glucose-lowering medication dose in response to glycemic improvements due to the meal replacement intervention. It is conceivable that the impact of our formula diet on the HbA1c reduction is underestimated due to this adjustment. Another limitation of our real-world study is that the participants of the control group were not randomly assigned. In one of our previously published studies (NCT02066831), we found dramatic negative effects on HbA1c and dropout rate (26%) for the participants of the control group [16]. This approach, without a randomized control group with standard care, was also conducted in other benchmark studies for formula diet trials, like the Counterbalance Study (CS) [14] and the Counterpoint Study (CP) [15]. Both of the studies with small sample sizes (*n* = 11–29) reduced HbA1c (CS: −1.4% and CP: −1.1% to −0.6%) similar, as it was shown in our study after eight weeks of intervention. Furthermore, when comparing the present study results with findings from other landmark studies (DIRECT and TeLiPro study [17,21]), one can see that an assignment to the control group with standard care is accompanied with serious and disadvantageous effects, such as high dropout rates or even an increase in HbA1c. These findings support our approach and study design.

In sum, individualized low-carbohydrate diets can produce clinically-relevant reductions in HbA1c after 12 weeks of intervention. Furthermore, body weight, fasting blood glucose, quality of life, eating behavior, and other cardiometabolic risk factors improved, although not all of the parameters showed statistically significant improvements. Moreover, the initiation of a weight loss program with one week of full meal replacement (three meals per day) resulted in a clinically relevant long-term HbA1c reduction, when compared to an observational control group that had standard care. Our practicable and real-world setting-based approach led to relevant long-term improvements that were comparable with procedures of bariatric surgery without adverse events or negative side-effects. These results support the therapeutic concept of low-carbohydrate diets by formula diets in patients with poorly controlled type 2 diabetes.

## Figures and Tables

**Table 1:** Baseline characteristics of the participants who finished the 12-week diet intervention.

	M-Group (*n* = 125)	S-Group (*n* = 122)	Control Group (*n* = 74)
Sex (% male)	46.4	52.5	52.7
Age (years)	60 ± 10	59 ± 9	60 ± 8
Weight (kg)	110 ± 24	107 ± 20	111 ± 21
BMI (kg/m^2^)	37.5 ± 7.6	36.1 ± 5.9	37.0 ± 6.7
HbA1c (%)	8.4 ± 1.1	8.4 ± 1.2	8.2 ± 1.2
Known diabetes duration (years)	9 ± 6	8 ± 7	11 ± 8 ^‡,ⱡ^
FBG (mg/dL)	181 ± 53	178 ± 63	179 ± 54
SBP (mmHg)	135 ± 17	134 ± 14	134 ± 13
DBP (mmHg)	82 ± 8	80 ± 8	81 ± 9
Total cholesterol (mg/dL)	200 ± 52	198 ± 43	194 ± 48
HDL (mg/dL)	46 ± 10	47 ± 11	47 ± 11
LDL (mg/dL)	118 ± 32	119 ± 37	117 ± 36
Triglyceride (mg/dL)	383 ± 586	220 ± 157	194 ± 113
TFEQ [cognitive control] (au)	10 (7; 13)	10 (7; 13)	7 (6; 8) ^ⱡⱡ, ‡‡^
TFEQ [suggestibility] (au)	7 (5; 10)	7 (4; 10)	5 (3; 6) ^ⱡⱡ, ‡‡^
TFEQ [hunger] (au)	6 (4; 9)	5 (3; 9)	5 (4; 8)
SF36 [physical health] (au)	42 (35; 50)	42 (34; 51)	40 (31; 52)
SF36 [mental health] (au)	49 (38; 57)	49 (32; 57)	39 (35; 42) ^ⱡⱡ,‡‡^

Shown are means ± SD, median (1st; 3rd quartiles) or percentages. ^ⱡⱡ^ CON vs. M-group, *p* < 0.01; ^ⱡ^ CON vs. M-group, *p* < 0.05; ^‡‡^ CON vs. S-group, *p* < 0.01; ^‡^ CON vs. S-group, *p* < 0.05; au, arbitrary units; FBG, fasting blood glucose; BMI, body mass index; DBP, diastolic blood pressure; HDL, high-density-lipoprotein; LDL, low-density-lipoprotein; SF36, short form-36; SBP, systolic blood pressure; TFEQ, three-factor eating questionnaire.

**Table 2:** Group comparison between S-group and M-group after 12 and 52 weeks (primary endpoints).

	12 Weeks			52 Weeks		
	S-Group (*n* = 122)	M-Group (*n* = 125)	*P*	S-Group (*n* = 111)	M-Group (*n* = 112)	*P*
Sex (% male)	52.5	46.4	0.374	50.4	46.3	0.593
Age (years)	59 ± 9	60 ± 10	0.966	59 ± 9	60 ± 10	0.523
Weight (kg)	103 ± 22	103 ± 23	0.333	98 ± 17	101 ± 23	0.245
BMI (kg/m^2^)	33.9 ± 5.6	35.1 ± 7.5	0.108	33.2 ± 5.1	34.8 ± 7.6	0.074
HbA1c (%)	7.5 ± 1.3	7.6 ± 1.1	0.539	7.6 ± 1.3	7.9 ± 1.4	0.085
Known diabetes duration (years)	7.7 ± 6.6	8.6 ± 6.4	0.265	7.3 ± 5.2	8.9 ± 6.6	0.053
FBG (mg/dL)	154 ± 54	157 ± 50	0.673	156 ± 51	165 ± 52	0.163
RR [syst] (mmHg)	128 ± 14	129 ± 16	0.404	128 ± 14	129 ± 13	0.507
RR [dia] (mmHg)	77 ± 8	79 ± 8	0.082	77 ± 8	78 ± 8	0.339
Total cholesterol (mg/dL)	191 ± 43	190 ± 38	0.829	198 ± 50	194 ± 48	0.571
HDL (mg/dL)	47 ± 10	46 ± 11	0.661	51 ± 36	47 ± 12	0.253
LDL (mg/dL)	116 ± 36	112 ± 31	0.357	120 ± 37	111 ± 33	0.054
Triglyceride (mg/dL)	193 ± 111	205 ± 193	0.564	190 ± 102	368 ± 534	0.025
TFEQ [cognitive control] (au)	13 (9; 16)	13 (9; 16)	0.590	13 (9; 16)	13 (9; 16)	0.704
TFEQ [suggestibility] (au)	5 (3; 8)	6 (3; 10)	0.313	6 (4; 8)	6 (4; 9)	0.189
TFEQ [hunger] (au)	3 (2; 6)	4 (2; 8)	0.131	3 (1; 6)	5 (2; 8)	0.034
SF36 [physical health] (au)	46 (38; 53)	46 (35; 52)	0.277	46 (37; 52)	42 (34; 52)	0.052
SF36 [mental health] (au)	51 (35; 58)	52 (38; 58)	0.330	49 (29; 56)	52 (37; 58)	0.074
No medication (%)	8.2	6.4	0.632	8.2	4.5	0.285
Metformin (%)	76.2	81.6	0.350	76.2	80.4	0.625
DPP4 inhibitors (%)	23.8	29.6	0.317	23.8	33.9	0.187
Sulfonylureas (%)	1.6	4.0	0.447	1.6	8.9	0.285
Glinides (%)	0	0	NA	0	2.7	0.622
Glitazone (%)	0	0	NA	0	0.9	0.990
Glucosidase inhibitors (%)	0	0	NA	0.9	0	0.990
GLP-1 receptor agonists (%)	9.0	11.2	0.674	9.0	11.6	0.661
Sodium-glucose co-transporter-2 (%)	0.8	0.8	0.990	0.9	0.9	0.990
Insulin (%)	18.9	13.6	0.302	18.9	15.3	0.140

Shown are means ± standard deviations, median (1st; 3rd quartiles) or percentages. Differences after 12 and 52 weeks between groups were analyzed using multivariable regression models adjusting for baseline values; au, arbitrary units; FBG, fasting blood glucose; BMI, body mass index; DBP, diastolic blood pressure; HDL, high-density-lipoprotein; LDL, low-density-lipoprotein; NA, not applicable; SBP, systolic blood pressure; SF36, short form-36; TFEQ, three-factor eating questionnaire; DDP4, dipeptidyl peptidase 4; GLP-1, glucagon-like peptide-1.

**Table 3:** Changes of anthropometric, clinical, pharmaceutical, and behavioral parameters (secondary endpoints).

	M-Group (*n* = 125)	S-Group (*n* = 122)	*p*
HbA1c (%)	8.4 ± 1.1	8.4 ± 1.2	
Δ HbA1c (%) 12 weeks	−0.84 [−1.08; −0.61] ***^,a^	−0.97 [−1.21; −0.74] ***^,a^	0.538
Δ HbA1c (%) 52 weeks	−0.55 [−0.80; −0.29] ***^,a^	−0.81 [−1.06; −0.55] ***^,a^	0.149
Weight (kg)	110 ± 24	107 ± 20	
Δ Weight (kg) 12 weeks	−6.93 [−8.08; −5.78] ***^,a^	−6.91 [−8.07; −5.76] ***^,a^	0.999
Δ Weight (kg) 52 weeks	−7.30 [−8.65; −5.95] ***^,a^	−7.45 [−8.80; −6.10] ***^,a^	0.615
BMI (kg/m^2^)	37.5 ± 7.6	36.1 ± 5.9	
Δ BMI (kg/m^2^) 12 weeks	−2.38 [−2.78; −1.98] ***^,a^	−2.35 [−2.75; −1.95] ***^,a^	0.911
Δ BMI (kg/m^2^) 52 weeks	−2.36 [−2.84; −1.88] ***^,a^	−2.50 [−2.98; −2.02] ***^,a^	0.536
FBG (mg/dL)	181 ± 53	178 ± 63	
Δ FBG (mg/dL) 12 weeks	−24 [−34; −13] ***^,a^	−25 [−36; −15] ***^,a^	0.791
Δ FBG (mg/dL) 52 weeks	−17 [−30; −5] **	−22 [−35; −10] ***^,a^	0.196
SBP (mmHg)	136 ± 17	134 ± 14	
Δ SBP (mmHg) 12 weeks	−5.6 [−8.7; −2.5] ***^,a^	−6.6 [−9.7; −3.5] ***^,a^	0.512
Δ SBP (mmHg) 52 weeks	−6.0 [−9.3; −2.7] ***^,a^	−5.8 [−9.1; −2.5] ***^,a^	0.858
DBP (mmHg)	82 ± 8	80 ± 8	
Δ DBP (mmHg) 12 weeks	−2.9 [−4.5; −1.3] ***^,a^	−3.0 [−4.6; −1.4] ***^,a^	0.371
Δ DBP (mmHg) 52 weeks	−3.7 [−5.6; −1.9] ***^,a^	−2.9 [−4.8; −1.0] **	0.992
Total cholesterol (mg/dL)	200 ± 52	198 ± 43	
Δ Total cholesterol (mg/dL) 12 weeks	−11.1 [−18.9; −3.3] **	−7.0 [−14.7; 0.8]	0.565
Δ Total cholesterol (mg/dL) 52 weeks	−8.0 [−17.3; 1.3]	0.1 [−9.3; 9.4]	0.396
HDL (mg/dL)	46 ± 10	47 ± 11	
Δ HDL (mg/dL) 12 weeks	−0.1 [−1.6; 1.4]	−0.1 [−1.6; 1.4]	0.908
Δ HDL (mg/dL) 52 weeks	0.9 [−4.0; 5.9]	4.5 [−0.5; 9.5]	0.248
LDL (mg/dL)	118 ± 32	119 ± 37	
Δ LDL (mg/dL) 12 weeks	−6.6 [−10.9; −2.3] **	−3.3 [−7.6; 1.0]	0.144
Δ LDL (mg/dL) 52 weeks	−7.6 [−12.8; −2.4] **	1.8 [−3.4; 7.0]	0.012
Triglyceride (mg/dL)	383 ± 586	220 ± 157	
Δ Triglyceride (mg/dL) 12 weeks	−186 [−268; −104] ***^,a^	−27 [−109; 56]	0.041
Δ Triglyceride (mg/dL) 52 weeks	−35 [−86; 17]	−31 [−83; 21]	0.865
TFEQ [cognitive control] (au)	9.7 ± 3.9	10.0 ± 4.3	
Δ TFEQ [cognitive control] (au) 12 weeks	2.5 [1.7; 3.3] ***^,a^	2.5 [1.7; 3.3] ***^,a^	0.847
Δ TFEQ [cognitive control] (au) 52 weeks	2.3 [1.5; 3.1] ***^,a^	2.2 [1.4; 3.0] ***^,a^	0.633
TFEQ [suggestibility] (au)	7.4 ± 3.8	7.0 ± 3.5	
Δ TFEQ [suggestibility] (au) 12 weeks	−0.8 [−1.3; −0.3] **	−0.8 [−1.4; −0.3] ***^,a^	0.686
Δ TFEQ [suggestibility] (au) 52 weeks	−0.8 [−1.3; −0.2] **	−0.9 [−1.4; −0.4] ***^,a^	0.342
TFEQ [hunger] (au)	6.3 ± 3.7	5.6 ± 3.3	
Δ TFEQ [hunger] (au) 12 weeks	−1.3 [−1.8; −0.7] ***^,a^	−1.3 [−1.9; −0.7] ***^,a^	0.586
Δ TFEQ [hunger] (au) 52 weeks	−1.1 [−1.7; −0.5] ***^,a^	−1.4 [−2.0; −0.8] ***^,a^	0.074
SF36 [physical health] (au)	42 ± 10	43 ± 10	
Δ SF36 [physical health] (au) 12 weeks	1.5 [−0.2; 3.2]	1.4 [−0.3; 3.1]	0.773
Δ SF36 [physical health] (au) 52 weeks	0.2 [1.4; 1.8]	1.2 [−0.4; 2.8]	0.150
SF36 [mental health] (au)	47 ± 13	45 ± 15	
Δ SF36 [mental health] (au) 12 weeks	0.6 [−2.0; 3.2]	1.2 [−1.5; 3.8]	0.953
Δ SF36 [mental health] (au) 52 weeks	−0.4 [−3.0; 2.2]	−1.4 [−3.9; 1.2]	0.272
No medication (%)	8.0	8.2	
Δ no medication (%) 12 weeks	−1.6	0	0.652
Δ no medication (%) 52 weeks	−3.5	−0.1	0.179
Metformin (%)	81.6	77.0	
Δ Metformin (%) 12 weeks	0	−0.8	0.660
Δ Metformin (%) 52 weeks	−1.2	0.5	0.942
DPP4 inhibitors (%)	28.8	24.6	
Δ DPP4 inhibitors (%) 12 weeks	0.8	−0.8	0.314
Δ DPP4 inhibitors (%) 52 weeks	5.1	0.6	0.377
Sulfonylurea (%)	6.4	4.1	
Δ Sulfonylurea (%) 12 weeks	−2.4	−2.5	1.000
Δ Sulfonylurea (%) 52 weeks	2.5	0.4	0.920
Glinides (%)	0	0	
Δ Glinides (%) 12 weeks	0	0	NA
Δ Glinides (%) 52 weeks	2.7	0.9	0.622
Glitazone (%)	1.6	0.8	
Δ Glitazone (%) 12 weeks	−1.6	0	0.428
Δ Glitazone (%) 52 weeks	−0.5	−0.8	1.000
Glucosidase inhibitors (%)	0	0.8	
Δ Glucosidase inhibitors (%) 12 weeks	0	0	NA
Δ Glucosidase inhibitors (%) 52 weeks	0	0.1	1.000
GLP−1 receptor agonists (%)	12.0	8.2	
Δ GLP−1 receptor agonists (%) 12 weeks	−0.8	0.8	0.855
Δ GLP−1 receptor agonists (%) 52 weeks	0.4	0.8	1.000
Sodium-glucose co-transporter−2 (%)	0.8	0.8	
Δ Sodium-glucose co-transporter−2 (%) 12 weeks	0	0	NA
Δ Sodium-glucose co-transporter−2 (%) 52 weeks	0.1	0.1	1.000
Insulin (%)	19.2	19.7	
Δ Insulin (%) 12 weeks	−5.6	−0.8	0.290
Δ Insulin (%) 52 weeks	−3.9	−0.8	0.256

Data are shown as mean ± SD and mean [95% CI] or % as appropriate; *** *p* < 0.001 vs. baseline; ** *p* < 0.01 vs. baseline; Superscript letter a represents significance after Bonferroni correction for multiple testing (*p* < 0.002). Differences in changes after 12 and 52 weeks between both groups were analyzed using multivariable regression models adjusting baseline values. au, arbitrary units; BMI, body mass index; DBP, diastolic blood pressure; SBP, systolic blood pressure; SF36, short form-36 questionnaire; FBG, fasting blood glucose; HDL, high-density-lipoprotein; LDL, low-density-lipoprotein; TFEQ, three-factor eating questionnaire; DDP4, dipeptidyl peptidase 4; GLP-1, glucagon-like peptide-1. NA, not applicable.

## Supplementary Materials

The following are available online at http://www.mdpi.com/2072-6643/10/8/1022/s1. Table S1. Baseline characteristics of the drop-outs. Table S2. Baseline antidiabetic drugs.
