# Supplementary material for: Individualized Meal Replacement Therapy Improves Clinically Relevant Long-Term Glycemic Control in Poorly Controlled Type 2 Diabetes Patients
Source: Nutrients. 2018 Aug 4;10(8):1022. doi: 10.3390/nu10081022 (PMC6115894; doi:10.3390/nu10081022)
Supplement: Supplementary file 1 [file nutrients-10-01022-s001.pdf]

Supplementary Table 1. Baseline characteristics of the drop-outs.

	M-group (n=34)	S-group (n=28)	Control group (n=44)
Sex (% male)	38.2	67.9	47.7
Age (years)	56 ±12	61 ±9	62 ±7 ł
Weight (kg)	112 ±19	113 ±23	106 ±20
BMI (kg/m²)	37.9 ±5.8	38.0 ±7.9	36.6 ±5.4
HbA_{1c} (%)	8.7 ±1.7	8.5 ±1.6	8.1 ±1.1
Known diabetes duration (years)	8 ±6	11 ±10	13 ±6 ł
FBG (mg/dl)	182 ±59	182 ±61	176 ±55
SBP (mmHg)	134 ±13	139 ±16	134 ±14
DBP (mmHg)	81 ±8	82 ±5	78 ±8
Total cholesterol (mg/dl)	207 ±43	207 ±36	190 ±42
HDL (mg/dl)	47 ±12	47 ±11	50 ±24
LDL (mg/dl)	130 ±33	120 ±31	113 ±33
Triglyceride (mg/dl)	406 ±604	243 ±182	216 ±84
TFEQ [cognitive control] (au)	10 (8; 12)	9 (6; 11)	7 (6; 8) łł, ‡‡
TFEQ [suggestibility] (au)	7 (5; 10)	7 (4; 10)	5 (3; 6) łł
TFEQ [hunger] (au)	7 (5; 8)	7 (4; 11)	5 (4; 6)
SF36 [physical health] (au)	41 (30; 48)	39 (30; 45)	37 (30; 47)
SF36 [mental health] (au)	49 (35; 56)	48 (33; 57)	38 (35; 42) łł, ‡‡

Shown are means ± standard deviations, median (1st; 3rd quartiles) or percentages. łł CON vs. M-group, p<0.01; ł CON vs. M-group, p<0.05; ‡‡ CON vs. S-group, p<0.01; Underlined symbols (‡, ł) represent significant differences after Bonferroni correction for multiple testing; au, arbitrary units; FBG, fasting blood glucose; BMI, body mass index; DBP, diastolic blood pressure; HDL, high-density-lipoprotein; LDL, low-density-lipoprotein; SBP, systolic blood pressure; SF36, short form-36; TFEQ, three-factor eating questionnaire

Supplementary Table 2. Baseline antidiabetic drugs.

	M-group (n=125)	S-group (n=122)	Control group (n=74)	P (M- vs. S-group)	P (M- vs. Control-group)	P (S- vs. Control-group)
No medication (%)	8.0	8.2	0	1.000		0.014
Metformin (%)	81.6	77.0	89.2	0.433	0.164	0.037
DPP4 inhibitors (%)	28.8	24.6	33.8	0.475	0.525	0.111
Sulfonylureas (%)	6.4	4.1	13.5	0.571	0.124	0.018
Glinides (%)	0	0	5.4	1.000	0.018	0.019
Glitazone (%)	2.0	0.8	1.4	1.000	1.000	1.000
Glucosidase inhibitors (%)	0	0.8	0	0.494	1.000	1.000
GLP-1 receptor agonists (%)	12.0	8.2	21.6	0.400	0.104	0.009
Sodium-glucose co-transporter-2 (%)	0.8	0.8	4.2	0.742	0.402	0.405
Insulin (%)	19.2	19.7	75.7[a, b]	1.000	<0.001	<0.001

Data are shown as percentages. Superscript letters a (M-group vs. control group) and b (S-group vs. control group) represent significant differences after Bonferroni correction for multiple testing; DDP4, dipeptidyl peptidase 4; GLP-1, glucagon-like peptide-1.
